# Overexpression of secretagogin inhibits cell apoptosis and induces chemoresistance in small cell lung cancer under the regulation of miR-494

**DOI:** 10.18632/oncotarget.2305

**Published:** 2014-08-04

**Authors:** Yifeng Bai, Yanqin Sun, Juan Peng, Hongzhan Liao, Hongyi Gao, Ying Guo, Linlang Guo

**Affiliations:** ^1^ Department of Pathology, Zhujiang Hospital, Southern Medical University, Guangzhou, China; ^2^ Department of Oncology, Sichuan Academy of Medical Sciences & Sichuan Provincial People's Hospital, Chengdu, China; ^3^ Department of Pathology, School of Basic Medicine Science, Guangdong Medical College, Dongguan, China; ^4^ Department of Pathology, the Third Affiliated Hospital Of Guangzhou Medical University, Guangzhou, China; ^5^ Department of Neurosurgery, Zhujiang Hospital, Southern Medical University, Guangzhou, China; ^6^ Department of Pathology, Guangdong Women and Children Hospital, Guangzhou, China; ^7^ Department of Organ Transplantation, Zhujiang Hospital, Southern Medical University, Guangzhou, China

**Keywords:** SCGN, miR-494, SCLC, chemoresistance

## Abstract

Secretagogin (SCGN) has recently been identified to play a crucial role in cell apoptosis, receptor signaling and differentiation. However, its clinical significance and functional roles in SCLC chemoresistance remain unknown. Here we examined the expression of SCGN in clinical samples from SCLC patients and evaluated its relation with clinical prognosis. Then up and down-regulation of SCGN were carried out in SCLC cell lines to assess its influence on chemoresistance. Furthermore, luciferase reporter assay was used to evaluate whether SCGN is a novel direct target of miR-494. Our results revealed that elevated expression of SCGN was correlated with the poorer prognosis of SCLC patients and the more significant correlation with chemosensitivity. We also found that knockdown of SCGN expression in H69AR and H446AR cells increased chemosensitivity via increasing cell apoptosis and cell cycle arrest of G0/G1 phase, while over-expression of SCGN reduced chemosensitivity in sensitive H69 and H446 cells. SCGN as a novel target of miR-494 by luciferase reporter assay, up-regulation of miR-494 can sensitize H69AR cells to chemotherapeutic drugs. These results suggest SCGN is involved in the chemoresistance of SCLC under the regulation of miR-494 and may be a potential biomarker for predicting therapeutic response in treatment SCLC.

## INTRODUCTION

Lung cancer is one of the leading malignant tumors in the world. Small cell lung cancer (SCLC) accounts for approximately 15% of all lung cancers at present. The extreme aggressiveness of SCLC is due to its early and widespread metastasis and rapid development of multidrug resistance (MDR) to chemotherapy [1]. The current front-line standard chemotherapy regimen for SCLC composed of etoposide or irinotecan plus cisplatin, but SCLC recurs shortly after the first successful treatment with MDR phenotype [2-4]. Therefore, chemoresistance has become one of the major problems in the chemotherapy, it is also an important clinical issue to cause poor prognosis of SCLC.

Secretagogin (SCGN) is a recent identified novel EF-hand calcium-binding protein with similarities to calmodulin and calbindin-D28K [5], it expresses mainly in normal endocrine tissues, including distinct brain regions as well as neuroendocrine cells of gastrointestinal tract and pancreas [6]. SCGN has also been reported to expresses highly in various neuroendocrine-derived tumors, such as neuroendocrine carcinoids and carcinoid metastases, lung neuroendocrine tumors, pancreas, prostate cancer, adrenal gland, pituitary adenomas and colon carcinomas [5-11]. Although recent studies have shown that SCGN is involved in cell cycle regulation, developmental processes, apoptosis, transcription and secretion control [12,13], the role of SCGN in SCLC chemoresistance has not been elucidated yet.

MicroRNAs (miRNAs) are a class of small non-coding RNAs of 18–24 nucleotides which are responsible for the post-transcriptional regulation of their target mRNAs [14]. The level of individual miRNA changes dramatically in different cell types and developmental stages, it participated in cell growth, differentiation and apoptosis [15-17]. The differential expression profiles of miRNAs from tumor and paratumor tissues show its important roles in tumor classification and prediction of therapy response [18,19]. Some studies have also explored that miRNAs are closely associated with the chemosensitivity or chemoresistance, such as miR-21, miR-195, miR-455-3p and miR-10a [20,21], they all contribute to the development of glioblastoma chemoresistance. In addition, miR-128-2, miR-200b and miR-494 were reported to be closely associated with lung cancer chemoresistance [22-25]. Our team found that miR-494 expresses much lower in H69AR cells than H69 cells by microarray and qRT-PCR [26]. However, there is still no report available about miR-494 functions in SCLC drug resistance until now.

To better understand the biological function of SCGN in SCLC, we detected the expression of SCGN in SCLC tissues and blood samples and evaluated the relevance of SCGN expression with clinical prognosis of SCLC patients. We then further investigated its potential role on chemoresistance by the cellular model of human SCLC resistant cell lines (H69AR and H446AR). In addition, luciferase reporter assay was used to confirm SCGN as a novel target of miR-494. Therefore, we conclude that SCGN may play a role in SCLC progression and chemoresistance under the regulation of miR-494.

## RESULTS

### SCGN expression in SCLC is correlated with clinical stage and survival

To investigate the clinicopathological features of SCGN expression in SCLC, immunohistochemistry staining was performed on 77 samples from SCLC patients. The slides were reviewed independently by 2 pathologists in a blinded manner according to the intensity of staining. Positively stained SCGN was mainly located in the cytoplasm of SCLC cells and appeared as light brown and brown particles. A sample was considered positive if more than 50% of the tumor cells retained nuclear staining, and 5 fields were randomly selected according to semiquantitative scales (high, 3; medium, 2; low, 1; no staining, 0), and only tumor cells were scored.

**Figure 1:**
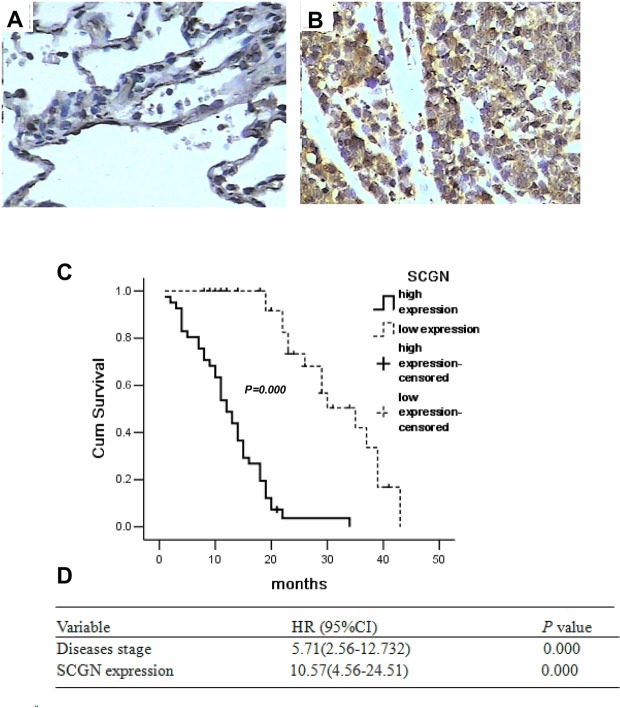
Expression of SCGN in diagnostic biopsy samples and the role of predicted clinical prognosis in SCLC A, Expression of SCGN in normal lung alveolar epithelium tissues by immunohistochemistry(400×). B, Expression of SCGN in small cell lung cancer biopsy samples by immunohistochemistry (400×). C, Survival differences between the different groups were statistically assessed by the Kaplan-Meier method and log-rank test. D, Cox regression analysis is performed using age (as a continuous variable), sex, stage and SCGN staining as input variables to identify SCGN and stage as independent predictors of survival. Values are presented as percentage of cell survival in high or low expression of SCGN in SCLC patients.

SCGN expression was localized in the cytoplasm of cancer cells (Fig.1B). No positive staining for SCGN was presented in the normal lung alveolar epithelium (Fig.1A). The positive rate of SCGN expression was 81.82% (63/77) in SCLC. Table 1 summarizes the relationship between SCGN expression and clinic pathological characteristics in SCLC patients. By Fisher's exact test, no significant difference was observed with respect to gender and age. Lower expression of SCGN was shown in limited disease-SCLC (LD-SCLC) than that in extensive disease-SCLC (ED-SCLC) (*P*=0.000). By univariate analysis, disease stage (*P*=0.000) and SCGN expression (*P*=0.000) were significantly associated with survival except gender and age. For overall survival, SCGN level was revealed to be closely correlates with significant overall survival of 77 SCLC patients via the Kaplan–Meier method (*P*=0.000, Fig.1C). Multivariate analysis were performed about SCGN expression (*P*=0.000) and disease stage (*P*=0.000) in SCLC patients (Table.1). SCGN expression was an independent predictor of survival with a hazard ratio of 10.57 and a 95% confidence interval ranging from 4.56 to 24.51 (Fig. 1D).

**Table 1:** Association of SCGN with clinical parameters

Patients Characteristics	SCGNexpression			
	-	+	χ2	P Value*
All cases (N=77)	14	63		
Age			0.416	0.519
≦56	6	33		
﹥56	8	30		
Gender			0.816	1.079
Male	5	35		
Female	9	28		
Disease stage			14.108	0.000
Limited disease (LD)	10	13		
Extensive-stage disease (ED)	4	50		
Median Survival (3-38months)			19.374	0.000
Survival	11	12		
Death	3	51		

* For analysis of correlation between of SCGN levels and clinical features, Fisher's Exact Test were used. Results were considered statistically significant at P <0 .05.

### SCGN expression is associated with SCLC chemoresistance

**Figure 2:**
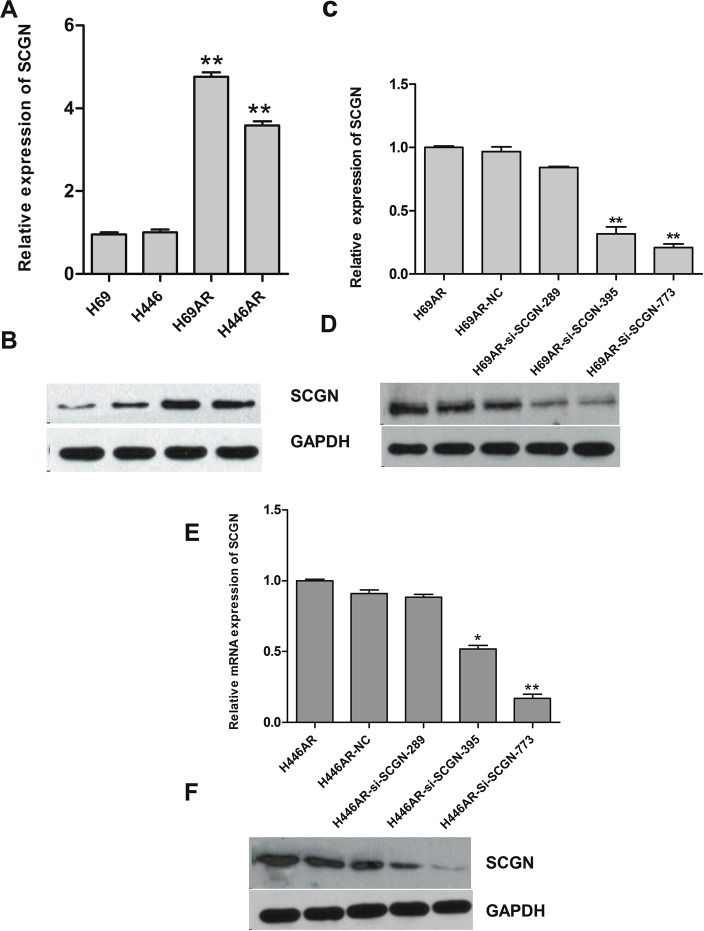
The expression of SCGN in SCLC cell lines The differential expression of SCGN was assessed in H69 and H446 cell lines compared with H69AR and H446AR cells by qRT-PCR (A) and Western blot (B). The expression of SCGN was detected when inhibition of SCGN by transfecting with three si-SCGNs (siRNA) or a scrambled negative control (NC) in H69AR cells by qRT-PCR(C) and Western blot(D). SCGN expression was examined after transfected with three si-SCGN (siRNA) or a scrambled negative control (NC) in H446AR cells by qRT-PCR (E) and Western blot (F). **P< 0.001,* *P*<0.05 compared with control.

**Figure 3:**
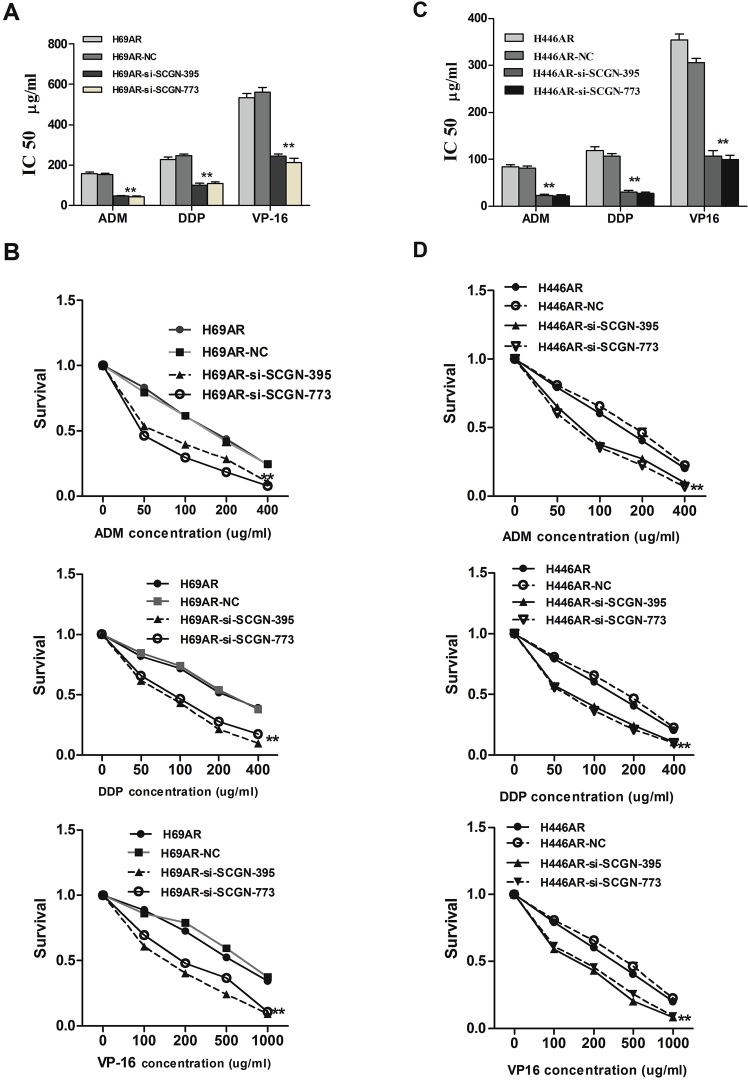
SCGN expression is associated with chemoresistance in SCLC The sensitivities (A,C) and survivals (B,D) of cells to chemotherapy drugs (ADM, DDP and VP-16) were measured after H69AR and H446AR cells transfected with SCGN-395,-773 or mock by CCK-8 assay. Values are presented as percentage of cell survival in drug-treated cells and untreated cells. **P< 0.001,* *P*<0.05 compared with control.

**Figure 4:**
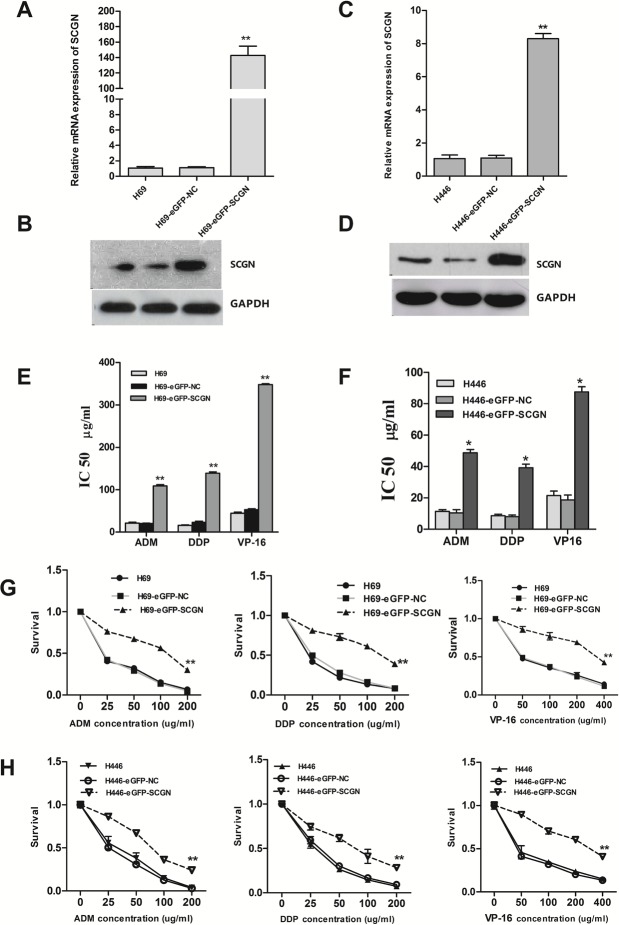
Enforced expression of SCGN in sensitive H69 and H446 cells increased its chemoresistance and survival after treated with chemotherapeutic drugs The mRNA (A,B) and protein (C,D) expression of SCGN in H69 and H446 cells after transfected with eGFP-SCGN-N1 and negative vector by qRT-PCR and Western blot. The drug sensitivity (E,F) and cell survival rate (G,H) were measured using CCK-8 assay. Values are presented as percentage of cell survival in drug-treated cells and untreated cells. **P< 0.001,* *P*<0.05 compared with control.

To investigate the effect of SCGN on SCLC chemoresistance, the differential expression of SCGN was detected in SCLC drug-resistant cells (H69AR and H446AR) and drug-sensitive cells (H69 and H446) by qRT-PCR and Western blot. The results showed that SCGN expression increased more significantly in H69AR and H446AR cells than that in H69 and H446 cells both at mRNA and protein level (Fig.2A&2B). To further investigate the effects of SCGN on chemoresistance of SCLC, we designed three siRNAs (SCGN-289, -395, -773) against SCGN to inhibit SCGN expression in H69AR and H446AR cells. SCGN-395 and SCGN-773 were selected for the followed study for their significant inhibition of SCGN expression both at mRNA and protein level (Fig.2C-2F, *P*=0.000). As expected, knockdown of SCGN resulted in the formation of sensitive phenotype. The cell survival and the IC_50_ values of SCGN-siRNAs transfected cells significantly decreased with treatment of chemotherapeutic drugs including ADM, DDP or VP-16 (Fig.3). In contrast, up-regulation of SCGN in H69 and H446 cells (Fig.4A-D) resulted in a significant increase of cell IC_50_ values, survival and the resistance to ADM, DDP or VP-16 (Fig.4E-H).

### Down-regulation of SCGN induces apoptosis of SCLC cells via anti-apoptotic gene Bcl-2

**Figure 5:**
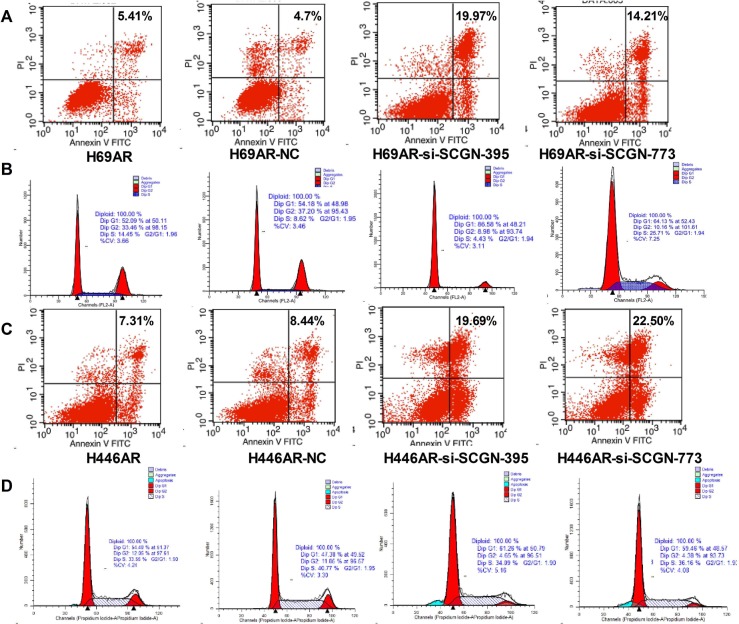
Inhibition of SCGN resulted in enhanced apoptosis rate and increased G0/G1 phase arrest in cell-cycle Cell apoptosis (A,C) and cell cycle (B,D) were assayed by flow cytometric analysis after H69AR and H446AR cells were transfected with SCGN-395,-773 or a negative control (NC).

**Figure 6:**
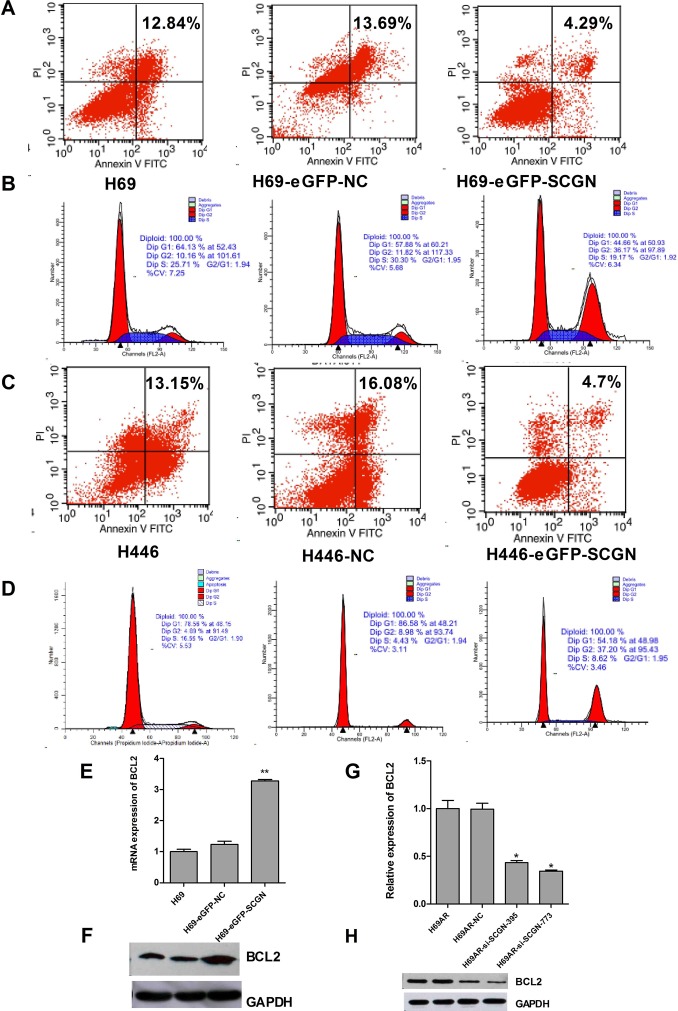
The role of SCGN in cell apoptosis and cell-cycle of sensitive SCLC cells Cell apoptosis (A,C) and cell cycle (B,D) were assayed by flow cytometric analysis after H69 and H446 cells were transfected with SCGN expression plasmid (eGFP-SCGN) or control plasmid (eGFP-NC). The expression of anti-apoptotic gene Bcl-2 was detected in mRNA (E,G) and protein (F,H) levels by qRT-PCR and Western blot after H69 and H69AR cells transfected with si-SCGN-395, -773, negative control (NC), eGFP-NC and eGFP-SCGN. **P< 0.001,* *P*<0.05 compared with control.

To investigate the possible mechanism of SCGN in SCLC chemoresistance, we found that down-regulation of SCGN resulted in increased cell apoptosis (Fig.5A&5C) and G0/G1 cell-cycle arrest (Fig.5B&5D) by flow cytometry analysis. Furthermore, knockdown of SCGN decreased anti-apoptotic gene Bcl-2 in mRNA and protein levels (Fig.6G&6H). On the other hand, enforced SCGN expression decreased cell apoptosis (Fig.6A&C) and the cell cycle arrest in G2/M (41.24%±6.56% vs. 8.86%±0.56%, P=0.000, Fig.6B&6D) by flow cytometry analysis. Meanwhile, over-expression of SCGN increased anti-apoptotic gene Bcl-2 both at mRNA and protein levels (Fig.6E&6F).

### SCGN is a direct target of miR-494

**Figure 7:**
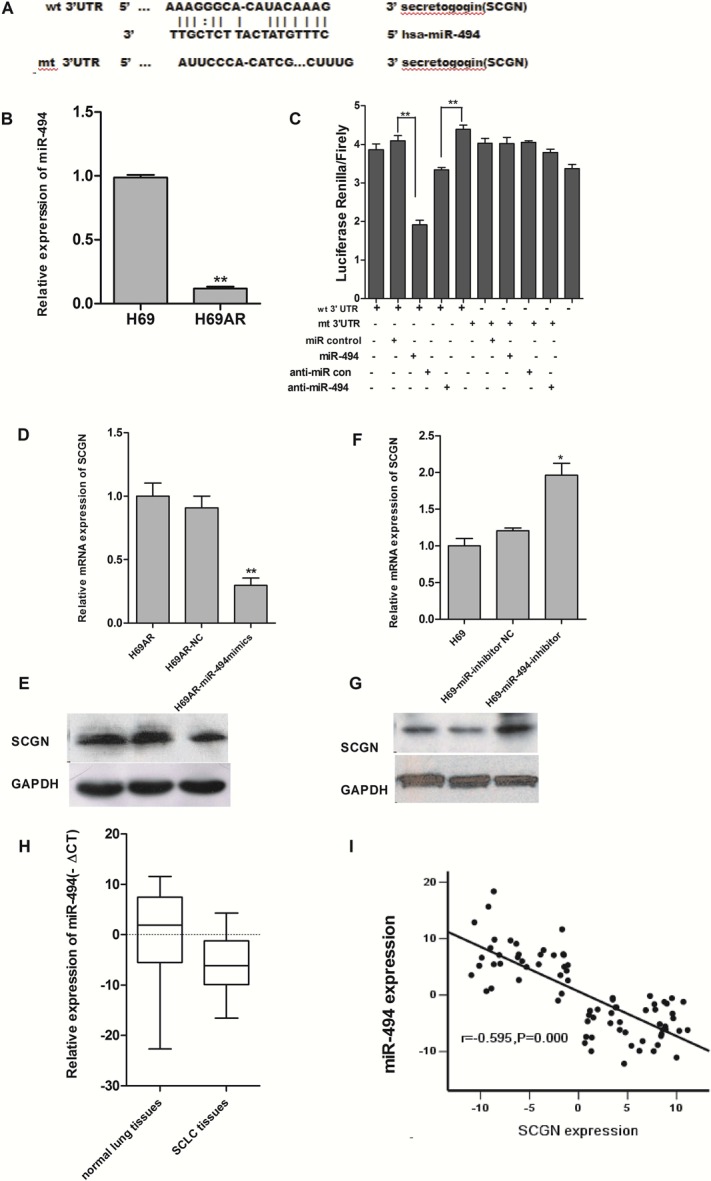
SCGN is a direct target of miR-494 A, Putative binding site of miR-494 in SCGN 3′UTR and the site of target mutagenesis are indicated. B, Expression of miR-494 in sensitive H69 cells and resistant H69AR cells by qRT-PCR. C, The dual luciferase assay was performed on H69AR cells transfected with luciferase construct alone or cotransfected with miR-494 mimics and miR-494 inhibitors. SCGN mRNA level (D) and protein level (E) were assessed 72 hrs after transfected with miR-494 mimics and negative control in H69AR cells. SCGN mRNA level (F) and protein level (G) were assessed 72 hrs after transfection of miR-494 inhibitors and negative control in H69 cells. H, Expression of miR-494 in SCLC FFPE tissues by qRT-PCR. I, The correlation of miR-494 and SCGN expression in FFPE tissues. **P<0.001,* *P*<0.05 compared with control.

By searching PicTar, TarScan and miRBase database, we found that 3′-UTR of human SCGN contains putative regions that match to the seed sequence of several miRNAs, including miR-494, which suggests a possible modulation of SCGN by miR-494 (Fig.7A). According to our previous studies, 6.93-fold decrease of miR-494 expression was revealed in H69AR cells by miRNA microarray. An 8.87-fold change of miR-494 in H69AR cells was further confirmed using qRT-PCR (Fig.7B).

To verify whether SCGN is a direct target of miR-494, we constructed luciferase reporters with wide-type (psiCHECK2-SCGN-mut-3′UTR) and mutated 3′-UTR (psiCHECK2-SCGN -3′ UTR) of SCGN. Both the wild-type and the mutant-type reporters were introduced into H69AR cells and co-transfected with miR-494 mimics or inhibitors. The Luciferase activity of SCGN was measured by the dual luciferase assays. As shown in Figure 7C, luciferase activity showed a significant decrease or increase as compared with either mutant or empty vector controls co-transfected with miR-494 mimics or inhibitors.

We further examined whether miR-494 can affect the endogenous expression of SCGN by up or down-regulation of miR-494 using miR-494 mimics or inhibitors respectively. SCGN expression at mRNA and protein levels decreased with transfection of miR-494 mimics in H69AR cells (Fig.7D&7E). Meanwhile, SCGN expression significantly increased in H69 cells transfected with miR-494 inhibitors (Fig.7F&7G). These findings suggest that miR-494 can directly target the 3′UTR of SCGN.

### Modulation of SCLC chemoresistance by SCGN is partly mediated under miR-494

**Figure 8:**
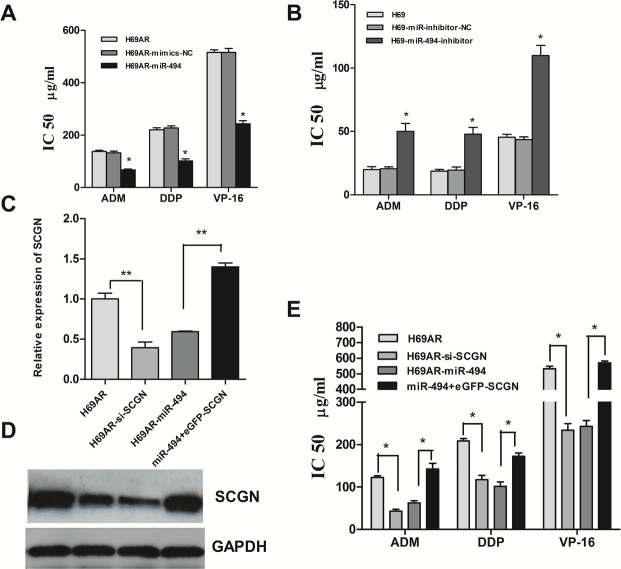
Modulation of chemoresistance by SCGN is partly mediated under miR-494 The sensitivities of cells to DDP, VP-16 and ADM after transfected with specific mimics (A) and inhibitors (B) of miR-494 or negative control. The expression of SCGN mRNA (C) and protein (D) were detected after transfected with siRNA, miR-494 or miR-494 plus eGFP-SCGN-N1 in H69AR cells. E, Cell sensitivities to chemotheraputic drugs were measured by CCK-8 assay in H69AR cells. **P< 0.001,* *P*<0.05 compared with control.

The results presented above indicated that miR-494 can regulate SCGN expression in SCLC cells. We then investigated whether miR-494 was also involved in SCLC chemoresistance. H69AR cells transfected with miR-494 mimics demonstrated an increasing sensitivity to ADM, DDP or VP-16 as compared with non-specific miRNAs (Fig.8A). In contrast, knockdown of miR-494 by transfected with miR-494 inhibitors in H69 cells can lead to decreasing sensitivity to ADM, DDP or VP-16 (Fig.8B).

Furthermore, we performed gain-of-function and loss-of-function studies to elucidate whether the effects of SCGN on chemoresistance were mediated by miR-494 in SCLC cells. Firstly, H69AR cells were transfected with SCGN-773 or miR-494 mimics. As shown in Figure 8C and 8D, knockdown of SCGN led to significantly increasing of chemosensitivity, which is similar to those induced by miR-494 mimics (Fig.8E, *P*<0.05). Subsequently, we evaluated whether ectopic expression of SCGN could rescue the suppressive effect of miR-494. H69AR cells were transfected with miR-494 mimics for 24 hours and followed by transfected with EGFP-SCGN, which encoded the full-length coding sequence without the 3′UTR region. The results showed that ectopic significant expression of SCGN can rescue drug resistance repression induced by miR-494 (Fig.8E, *P* <0.05).

### MiR-494 expression level shows a negative correlation with SCGN in SCLC FFPE tissues

We have showed SCGN was overexpressed in SCLC FFPE tissues and miR-494 can modulate SCGN expression in SCLC cells. The next question is whether there is any relationship between SCGN expression and miR-494. To address this issue, we extracted total RNA from the samples and analyzed miR-494 expression using qRT-PCR. Consistent with the data obtained from SCLC cell lines, higher expression level of miR-494 was showed in SCGN-negative specimens than in SCGN-positive SCLC tissues The results suggest that miR-494 was negatively correlated with SCGN expression (Fig.7H and 7I).

### Circulating SCGN is negatively correlated with miR-494 as a predictive marker of chemosensitivity in SCLC patients

**Figure 9:**
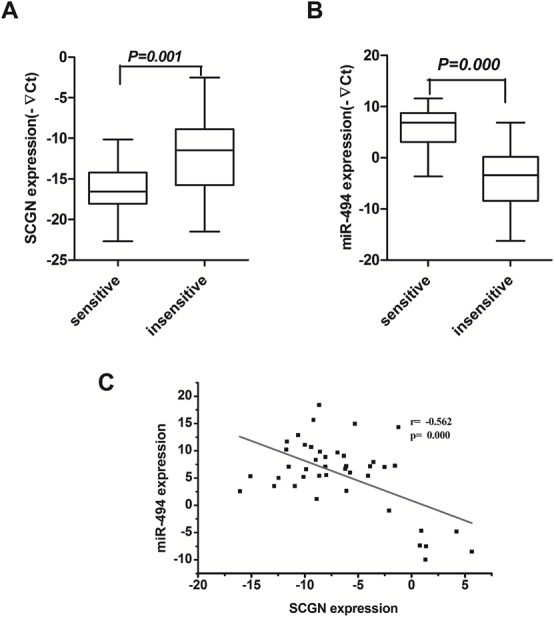
Blood SCGN acts as a predictive marker of chemosensitivity in SCLC and inversely correlates with miR-494 Expression of SCGN (A) and miR-494 (B) were detected in 18 chemosensitive cases and 24 chemoresistance cases by qRT-PCR. C, An inverse correlation between miR-494 and SCGN mRNA levels is shown in SCLC blood samples.

To evaluate the significance of the circulating SCGN level in chemosensitivity, 42 blood samples from the 77 patients mentioned above were collected from SCLC patient before and after chemotherapy. The expression of SCGN and miR-494 in blood samples were detected by qRT-PCR. The results show that SCGN is significantly lower in 18 sensitive cases to chemotherapy than that in 24 resistant cases (Fig.9A, *P*=0.001), while miR-494 is significantly higher in sensitive cases than refractory cases (Fig.9B, *P*=0.000). Figure 9C shows an inverse correlation (2-tailed Pearson correlation, r=-0.562, P=0.000) between SCGN and miR-494 expression at mRNA level in scatter diagram. SCGN level is closely associated with response to chemotherapy in SCLC (Supplementary Table S1). The results suggest that SCGN might be a predictive marker for chemosensitivity in SCLC.

## DISCUSSION

In this study, we reported the expression of SCGN and miR-494 in SCLC and its association with chemoresistance and clinical prognosis. We firstly analyzed the expression of SCGN in human SCLC tissues and blood samples and found that SCGN expression level significantly correlated with clinical stage and overall survival of the SCLC patients. Our data indicate that the expression levels of miR-494 and SCGN independently predict sensitivity of SCLC chemoresistance and disease outcome. Of note, the 42 blood samples are derived from the same patients of the FFPE samples, this increases the value of our work, in contrast to the similar studies with respective independent samples source. Most interestingly, recent study shown that SCGN may be one of genes involved in tumorgenesis and progression in Central Neurocytomas (CNs) [27]. In clear-cell renal cell carcinomas, expression of SCGN is associated with a high metastasis rate [13]. So far, the role of SCGN in SCLC have not been previously clarified. Therefore, our research may provide independent predictive indicator for SCLC patients.

Although SCGN gene is shown to be involved in various cellular processes including cell differentiation and proliferation [5,6], little is known regarding the role of SCGN genes in apoptosis and chemoresistance of SCLC. According to our data above, we have successfully established a H69AR-si-SCGN cell line with SCGN knockdown and a H69AR cell line with SCGN coordinated up-regulation. By CCK-8 assays, we found that knockdown of SCGN significantly enhanced chemosensitivity in H69AR-si-SCGN cells as compared to the H69AR cells. In contrast, over-expression of SCGN via stable transfect resulted in resistant phenotype of H69 cells. Therefore, our findings suggest SCGN may play an important role in chemoresistance of SCLC.

Based on the high level of SCGN expression in SCLC multidrug-resistant cells, we further evaluate the clinical significance of SCGN level in blood samples from clinical patients. According to the results, SCGN level in chemosensitive patients after given chemotherapeutic treatment is significantly lower than that before given chemotherapeutic treatment. Meanwhile, SCGN in refractory patients is slightly lower after given chemotherapeutic treatment than that before given chemotherapeutic treatment. Furthermore, compared with refractory patients, the decrease of SCGN circulating levels in chemosensitive patients was much more obvious, and the difference between these two groups shows significant. These findings suggest that the blood SCGN level could be a predictive marker of SCLC chemoresistance.

Bcl-2 family proteins have been clarified to participate tumorgenesis and chemoresistance in many tumors by researchers[33,37-40]. Based on the consideration above, we analyzed the expression of anti-apoptotic protein Bcl-2 and the effect of SCGN on cell apoptosis as well as cell cycle. Our results show that one reason of the resistant phenotype in H69AR cells may be that SCGN decreased drug-induced apoptosis and increased G2/M phase cell-cycle arrest via Bcl-2-regulated apoptosis pathway. Therefore, combined with the clinic-pathological information of patients, Bcl-2 may be another predictive marker of SCLC chemoresistance.

MiRNAs represent a novel class of genes that function as negative regulators of gene expression. Recently, miRNAs have been implicated in several cancer cells proliferation and chemoresistance [28,29]. However, aberrant miRNA expression and its chemoresistance in human SCLC have not been well documented. Here, we showed that miR-494 was down expression in H69AR cells. Using three prediction algorithms in the miRNAs database, miR-494 was predicted to have a potential interactive site at the 3′UTR of SCGN mRNA. Forced expression of miR-494 inhibited SCGN expression in resistance H69AR cells and rendered it resistant to chemotherapy, whereas knockdown of miR-494 elevated SCGN expression and overrode drug sensitive in H69 cells. Dual luciferase assays to verify SCGN as a direct target of miR-494. We also found that SCGN was widely over-expressed and inversely correlated with miR-494 in the blood samples and tissues from the SCLC patients. MiR-494 has recently been reported to suppress cell proliferation in lung cancer, cholangiocarcinoma and gastrointestinal stromal tumor cells [30-33]. Some genes such as IGF2BP1, CDK6, PTTG1, PTEN, p190B, and SMAD3 were identified direct targets of miR-494[30-36]. In our study, we firstly clarify the role of miR-494 in human SCLC chemoresistance by direct targeting of SCGN. The results indicate that high levels of miR-494/SCGN expression are associated with chemoresistance and predict SCLC patients' clinical outcome with the clinicopathological data. According to our data, we conclude that miR-494/SCGN may be involved in chemoresistance of SCLC through Bcl-2 pathway.

In conclusion, our study showed that SCGN level is closely associated with clinical stage, chemoresistance and overall survival in patients with SCLC. These findings suggest SCGN may serve as a biomarker predictive for chemoresistance and prognostic for survival of SCLC patients. Furthermore, we demonstrated that SCGN-mediated chemoresistance may be through Bcl-2 pathway and was regulated by miR-494. This study provides a novel mechanism of chemoresistance mediated by SCGN, suggesting that it may be a candidate target for developing therapeutic strategy to overcome drug resistance.

## MATERIALS AND METHODS

### Clinical samples

A total of 77 formalin-fixed, paraffin-embedded (FFPE) tissues and 42 blood samples out of the 77 cases were obtained from different patients who had received chemotherapy or bronchofiberscopy (BF) or biopsy for SCLC between the period 2008.01 and 2011.08, they all received care and follow-up in Southern Medical University affiliated Zhujiang Hospital (Guangzhou). The research protocols were approved by the Ethics Committee of Zhujiang Hospital. Samples were divided into “sensitive” (complete response or partial response) and “insensitive” (stable disease or progressive disease) groups according to the patient's responses assessed using medical image analysis and detection of serum tumor markers after 4 or 5 cycles of etoposide-based chemotherapy.

### Cell culture

Human SCLC cell line NCI-H69, NCI-H446 and the drug-resistant subline NCI-H69AR were purchased from the American Type Culture Collection (ATCC, USA) and maintained in RPMI 1640 medium contain in L-Glutamine with 10% and 20% fetal calf serum respectively in an incubator at 37°C with 5% CO_2_. The adriamycin-resistant NCI-H446 cell line (NCI-H446AR) was obtained by culturing these cells in gradually increasing doses of adriamycin up to 0.8 uM after a total of 14 months in our laboratory. The drug-resistant cells were maintained in drug-free medium for at least 2 weeks before any experiment.

### Establishment of H446AR cell line

H446AR cell line was obtained by culturing the NCI-H446 cells in gradually increasing doses of ADM. After 8 months, cells which grew in 0.4 uM ADM were obtained. After a further 6 months, cells which grew in 0.8 uM ADM were obtained. This cell line has been designated H446AR and has been maintained by alternate feedings with drug-free medium or medium containing 0.8 uM ADM. The stability of the resistant phenotype was determined by culturing continuously in medium with either 0.8 uM ADM or no drug and assessing relative resistance after various periods of time up to 5months. The process is established by referring establishment of H69AR (CANCER RESEARCH 47, 2594-2598, May 15, 1987).

### Cell transfection

Cells were transiently transfected with 100nmol/L of miR-494 mimics, inhibitors and miRNA negative control (miR-NC) (Bioneer, Korea), or 60nmol/L small interfering RNA (siRNA) specific to SCGN, scrambled siRNA negative control (NC) (Genepharma, Shanghai, China) by using Lipofectamine 2000 and OPTI-MEM I (Invitrogen). For stable transfection, SCGN expression plasmid (SCGN-eGFP-N1-1) and eGFP-N1 empty plasmid were transfected into NCI-H69 and NCI-H446 cells by using Lipofectamine 2000. Positive transfectants were selected in 500 ug/ml G418 (Calbiochem).

### RNA isolation and quantitative reverse transcription-PCR

Total RNA, including miRNAs, was isolated from cell lines, blood and FFPE tissues using TRIzol (Invitrogen), miRNeasy kit (Qiagen) and miRNeasy FFPE Kit (Qiagen) according to the manufacturer's instructions. cDNA synthesis was carried out according to PrimeScript RT reagent Kit (Takara, Dalian, China). The miRNA sequence-specific reverse transcription qRT-PCR for miR-494 and endogenous control U6 were performed according to Hairpin-it^TM^ miRNAs qRT-PCR quantization kit and U6 snRNA real-time PCR normalization kit (GenePharma, Shanghai, China). GAPDH or U6 snRNA was used as an endogenous control.

### Western blot analysis

Protein lysates were separated by 12% SDS-PAGE, and electrophoretically transferred to PVDF (polyvinylidene difluoride) membrane (Millipore). Then, the membrane was incubated with rabbit anti-human SCGN monoclonal antibody (Santa Cruze, USA) and mouse anti-human monoclonal antibody Bcl-2 (Abcam, Cambridge, MA, USA) at 4°C overnight. followed by HRP (horseradish peroxi-dase)-labeled goat anti-mouse or anti-rabbit IgG (Santa Cruz Biotechnology) and detected by chemiluminescence. Glyceraldehyde-3-phosphate dehydrogenase (GAPDH) was used as a protein-loading control. The intensity of protein fragments was quantified with the Quantity One software (4.5.0 basic, Bio-Rad).

### Cell counting kit-8 (CCK-8) assay

Cells were plated in 96-well plates at 5×10^3^ cells per well. After transient transfection or adherence of stable transfected cells, cells were treated with drugs for 24h. A total of three chemotherapy drugs [Cisplatin (DDP; Shandong, China), Etoposide (VP-16; Jiangshu, China), Adriamycin (ADM; Jiangshu, China)] were used. The absorbance at 450nm was measured after incubation with 10 μl of CCK-8 reagent (Dojindo, Kumamato, Japan) for 4h. The cells incubated without drugs were set at 100% survival and were used to calculate the concentration of each chemotherapeutic drug IC_50_. The assay was conducted in five replicate wells for each sample and three parallel experiments were performed.

### Flow cytometric analysis

Cells were treated with drugs for 24h after transfection, and then collected for apoptosis and cell-cycle assay. Cell apoptosis assay was performed by using AnnexinV/propidium iodide detection kit (Keygene, Nanjing, China). For cell-cycle assay, the cells were collected and fixed in 70% ethanol at 4°C for 16h and then stained with propidium iodide.

### Luciferase reporter assay

Cells were seeded in a 48-well plate and cotransfected with 200 ng of either pcDNA/miR-494 or pcDNA/miR-NC vectors, and 10 ng of pLUC vectors containing firefly luciferase reporter gene, as well as the 3′-UTR of SCGN gene. Cells were harvested for luciferase activity assays 48 hours after transfection. A luciferase assay kit (Promega, Madison, WI) was used according the manufacturer's protocol.

### Immunohistochemistry staining

Formalin-fixed, paraffin-embedded tissues of SCLC clinical patients samples were sectioned at 4 mm thickness and analyzed for SCGN (1:100, Sigma) expression. Visualization was achieved using the EnVision peroxidase system (Dako). A sample was considered positive if more than 50% of the tumor cells retained nuclear staining, and 5 fields were randomly selected according to semiquantitative scales. The intensity of staining was scored manually (high, 3; medium, 2; low, 1; no staining, 0) by 2 independent experienced pathologists, and only tumor cells were scored. Negative controls were performed by replacing the primary antibodies stated above with PBS. Positively stained SCGN was mainly located in the cytoplasm of SCLC cells and appeared as light brown and brown particles.

### Statistical analysis

All experiments were run in triplicate. Data are represented as Mean ± SD. All statistical analyses were carried out with SPSS 13.0 software. Possible differences between groups were analyzed using Student's t test or one-way ANOVA. The association between SCGN expression and clinical features were analyzed by Fisher's exact test. Survival curves were obtained by Kaplain-Meier method. Prognostic factors were examined by univariate and multivariate analyses (Cox proportional hazards model). *P*<0.05 was considered significant.

## SUPPLEMENTARY INFORMATION TABLE


