# Supplementary material for: Overexpression of secretagogin inhibits cell apoptosis and induces chemoresistance in small cell lung cancer under the regulation of miR-494
Source: Oncotarget. 2014 Aug 4;5(17):7760–75. doi: 10.18632/oncotarget.2305 (PMC4202159; doi:10.18632/oncotarget.2305)
Supplement: Supplementary file 1 [file oncotarget-05-7760-s001.pdf]

Overexpression of secretagogin inhibits cell apoptosis and induces chemoresistance in small cell lung cancer under the regulation of miR-494

Supplementary Information

Supplementary Table S1: Association of SCGN mRNA level in blood with clinical parameters in SCLC patients.

Patients Characteristics	SCGN expression		
	Low	High #	*P* Value*
All cases (*N=42*)	7	35	
Age			0.099
＜56	5	12	
≥56	2	23	
Gender			0.668
Male	4	24	
Female	3	11	
Disease stage			0.009
Limited disease (LD)	5	6	
Extensive-stage disease (ED)	2	29	
Response to chemotherapy			0.002
Response	6	7	
Refractory	1	28	
Median Survival (3-38months)			0.005
Survival	6	9	
Death	1	26	

#The median expression level was used as the cutoff. Low expression of SCGN in 7 patients was classified as values of $2^{-\Delta\Delta ct}$ below 1.0.High SCGN expression in 35 patients was classified as values of $2^{-\Delta\Delta ct}$ above 1.0.

*For analysis of correlation between of SCGN levels and clinical features,Fisher's Exact Test were used. Results were considered statistically significant at P <0 .05.
